# Editorial: Targeting ion channels for drug discovery: emerging challenges for high throughput screening technologies

**DOI:** 10.3389/fnmol.2024.1414816

**Published:** 2024-05-31

**Authors:** Ciria C. Hernandez, Luis E. Gimenez, Tim Strassmaier, Marc Rogers, Jean-Marc Taymans

**Affiliations:** ^1^Life Sciences Institute, University of Michigan, Ann Arbor, MI, United States; ^2^Nanion Technologies Inc., Livingston, NJ, United States; ^3^Albion Drug Discovery Services Ltd., Cambridge, United Kingdom; ^4^Univ. Lille, Inserm, CHU Lille, UMR-S 1172 - LilNCog - Lille Neuroscience & Cognition, Lille, France

**Keywords:** ion channel, high throughput screening (HTS), drug discovery and development, automated patch clamp (APC), channelopathy

Cellular functions are regulated by complex signaling networks that determine gene expression and cellular behavior. Membrane receptors transmit various types of information from the external environment to be processed at the membrane, cytoplasmic, or nuclear level (Ullo and Case, 2023). Among these receptors are the ligand and voltage-gated ion channels, which are highly regulated proteins that control ion flow and cellular excitability (Catterall and Swanson, 2015). Some ion channels are also membrane “sensors” like receptors, such as ASIC channels (pH) and TRPx receptors (Storozhuk et al., 2021; Zhang et al., 2023), triggering cellular responses in excitable tissues, such as neurons and muscle cells (Hille, 1978). Dysregulation of ion channels can lead to a wide range of disorders, including neuropathies, cardiac arrhythmias, muscle disorders, and metabolic diseases (Harraz and Delpire, 2024). With over 1.5% of the human genome represented by ion channels and a sizable (15 to 18%) proportion of small molecule drugs focusing on either sensor-, voltage-, or ligand-gated ion channels (Santos et al., 2017), this protein class is the second-largest category of pharmacologically targetable proteins after G protein-coupled receptors (Kaczorowski et al., 2008; Alexander et al., 2019), highlighting their clinical potential.

The first ion channel-targeting drugs date back to the last decade of the 19^th^ century with the discovery of cocaine-derived amino ester Na^+^ channel blockers leading to the discovery of the topical anesthetic lidocaine (c. 1943) and the class Ia antiarrhythmic procainamide (Cox, 2014). These discoveries paved the way for the FDA-approved drugs currently used in the clinic, including L-type voltage-gated Ca^2+^ channel blockers for treating hypertension (e.g., amlodipine, nifedipine, and verapamil), stroke (e.g., nimodipine), and arrhythmias (e.g., verapamil and diltiazem), and N- and T-type Ca^2+^ channel blockers for analgesic or anti-convulsant activity, and anti-convulsant Na^+^ channel inhibitors and KCNQ2/Kv7 K^+^ channel openers (e.g., retigabine) for treating epilepsy, to name a few.

The field has undergone considerable progress in the past 15 years, fueled by advancements in rodent and human genetic target detection and validation, structure-based drug design, high throughput computational modeling and disease modeling at the cellular level. With the acknowledgment of ion channels as quintessential drug targets, a deeper understanding of their pharmacology and structure has paved the way for substantial breakthroughs. Functional assays and instrumentation have significantly improved, leading to high-throughput screening (HTS) technologies designed explicitly for ion channels. Nonetheless, these targets present a challenging puzzle, and developing a successful *in vitro* drug profile for ion channel modulators remains a formidable goal. The current Research Topic showcases cutting-edge HTS technologies for various classes of ion channels, offering a glimpse into the forefront of research and innovation in this dynamic and vital field.

The first part of this Research Topic is devoted to the optimization of automated patch clamp (APC) and optogenetic screening platforms to accurately capture the nuances of ion channel activity and modulation. Ridley et al. focus on acid-sensing ion channels (ASIC), ligand-gated receptors playing a crucial role in detecting inflammation, tissue injury, and hypoxia-induced acidosis, and are thus considered promising targets for drug discovery in areas such as pain, oncology, and ischemia. The research outlines the development, optimization, and validation of various fast perfusion protocols for studying ligand-gated ion channels across multiple APC platforms using the hASIC1a channel as a case study. On the other hand, Rapedius et al. challenge the use of fluoride as a seal enhancer in APC experiments, which has been a longstanding practice for achieving optimal voltage control due to the criticality of seal and access resistance. The authors have developed APC recording substrates for high-throughput fluoride-free recordings on a 384-well APC system, achieving success rates exceeding 40% for GΩ seals.

The studies brought by Govorunova et al. and Borja et al. describe the combination of high-throughput, optical systems and reagents capable of collecting information-dense data from patch clamp and optogenetic assays in both heterologous and induced pluripotent stem cell-derived models. Govorunova et al. utilize a high-throughput APC platform to demonstrate the potential for discovery of new cation- and anion-selective channelrhodopsins (ChRs), discussing the advantages and limitations of employing the APC platform for ChR-coupled HTS. Borja et al. introduced Swarm™, a custom-designed all-optical instrument addressing the critical need for a compatible technology to enhance the application of optogenetic assays in drug screening. With variable-intensity blue-light optogenetic stimulation, Swarm™ facilitates membrane depolarization and enables fluorescence detection of changes in membrane potential or calcium levels.

Finally, Rosholm et al. review the state of APC techniques applied to the study of pluripotent stem cells (PSC). In particular, the authors posit that high-throughput electrophysiological profiling of human PSCs as cells differentiate will find application in personalized medicine based on stem-cell therapeutics.

In contrast to the high-throughput approaches described above, Mayar et al. showed the direct effect of cannabinoids on hyperpolarization-activated cyclic-nucleotide-gated (HCN1) channels using the two-electrode voltage clamp technique. The authors propose that cannabinoid-related drugs can directly affect channel function by bypassing GPCR activation, which may impact the use of these drugs as therapeutic entities.

Two additional articles focused on developing more effective drugs. The first review by Clement et al. discussed the neurological roles of ATP-sensitive potassium (KATP) channels and highlighted the potential for blocking the Kir6.1/SUR2B subtype as a promising approach to developing drugs to treat migraines. On the other hand, Melancon et al. delve into the limitlessness of chemical space and the intricacies of ion channels, presenting hurdles in identifying potential drug candidates and providing a comprehensive overview of cutting-edge computational chemistry methodologies for screening extensive compound libraries.

Further, in a cutting-edge study, Tsortouktzidis et al. used CRISPR activation and interference/inhibition systems (CRISPRa/i) to target promoter sequences and modulate gene expression in a highly specific manner. The study focused on the *Cacna1h* gene, which encodes the low-voltage-activated T-type calcium channel CaV3.2 linked to channelopathies associated with epileptic seizures. The researchers demonstrated the feasibility of a newly developed CRISPRa/i toolbox in manipulating the promoter activity of Cacna1h in various cell types, providing a promising approach to studying the functional effects of gain-of-function or loss-of-function variants in the *Cacna1h* gene. Following this channelopathy topic, it remains crucial to accurately profile the functional effects of patient-derived and disease-linked ion channel mutations and polymorphisms to determine their pathogenic potential. Accordingly, Ye et al. conducted a comprehensive analysis of 11 *de novo* KCNQ2 Kv7.2 variants linked to epileptic encephalopathies using manual patch clamp. The team used different combinations of homomeric KCNQ2 and heteromeric KCNQ2/KCNQ3 channels in a semi-high throughput manner.

In conclusion, the work presented in this Research Topic on ion channels as drug targets has highlighted novel approaches for drug screening and strategies to improve ion channel drug targeting. Technological progress in the field will no doubt spawn further work to discover drugs to modulate this yet underexploited class of drug targets, the ion channels.

## Author contributions

CH: Conceptualization, Investigation, Project administration, Supervision, Validation, Writing – original draft, Writing – review & editing. LG: Conceptualization, Investigation, Project administration, Supervision, Validation, Writing – original draft, Writing – review & editing. TS: Conceptualization, Investigation, Project administration, Supervision, Writing – original draft, Writing – review & editing. MR: Conceptualization, Investigation, Project administration, Supervision, Writing – original draft, Writing – review & editing. J-MT: Conceptualization, Investigation, Project administration, Supervision, Writing – original draft, Writing – review & editing.
